# Identification of the initial water-site and movement in *Gleditsia sinensis* seeds and its relation to seed coat structure

**DOI:** 10.1186/s13007-021-00756-z

**Published:** 2021-05-25

**Authors:** Mingwei Zhu, Song Dai, Qiuyue Ma, Shuxian Li

**Affiliations:** 1 Collaborative Innovation Center for Sustainable Forestry in Southern China, College of Forestry, Nanjing Forestry University, Nanjing, 210037 China; 2 Institute of Forestry Scientific Research and Technology Extension, Tongren Academy of Sciences, Tongren, 55430 China; 3 Institute of Leisure Agriculture in Jiangsu Academy of Agricultural Sciences, Nanjing, 210014 China

**Keywords:** Hardseededness, Leguminous seeds, Hot water treatment, Micropyle, MRI

## Abstract

**Background:**

Water uptake is essential for seed germination. However, *Gleditsia sinensis* seeds have a water-impermeable seed coat, which is beneficial for its adaption to the environment, but prohibits its germination without treatment. This feature may be associated with the structure of the seed coat. Thus, the aim of this research was to identify and describe the initial water uptake site and water movement and to determine the relationship between seed coat structure and water absorption.

**Results:**

A water temperature of 80 °C was optimal to break the hardseededness of *G. sinensis* seeds. Scanning electron microscopy (SEM) images revealed that the seed coat consisted of a palisade layer and light line that can hinder water entry into the seed. Also, a structure of vascular bundles existed in the hilar region. Hot water treatment caused the tightly closed micropyle to open and the cavity beneath it expanded; the layer of palisade cells in the lens was raised. The embryo dye-tracking tests showed that the radicle tip was the initial region to be stained red. After staining for 24 h, the red-stained area on the vascular bundle side of cotyledon was more extensive than that on the other side. Further studies by MRI maps indicated that the micropyle was the initial site for water imbibition. Some water then migrated along the space between the seed coat and the endosperm to the chalazal; simultaneously, the rest of the water reached the embryonic axis and spread into cotyledons. The maps of 20–24 h showed that water was unevenly distributed within the cotyledons in a way that the edge parts were more hydrated than the center. Blocking tests showed that the hilar region was the initial and an important region during seed imbibition. The medial region and chalazal portion were capable of imbibing water when the hilar region was blocked, but water absorption was later and slower than that through the hilar region.

**Conclusion:**

MRI technology provides a promising and non-invasive technique to identify the water gap and the path of water movement in the seed. Combined with the results of SEM, the relation between seed coat and its imbibition can be inferred.

## Background

Imbibition is the initial and most important stage of seed germination. Water is essential for the resumption of metabolic activity in the seed for processing starch, proteins, enzymes, and other macromolecules, which provide the necessary nutrients for germination. Therefore, the permeability of the seed coat to water is critical for seed germination. However, in many species, especially members of the Leguminosae, the structure of the seed coat hinders water absorption. Many studies have been conducted on the mechanism of water absorption by Leguminosae seeds. Studying water uptake and distribution during the rapid initial uptake stage is essential to understanding the process of seed germination [1]. A variety of methods, such as physically blocking different regions of the seed or the dying test, can be used to identify the route of water entry into the seed. However, these methods are destructive and cannot reflect the process of water uptake in real time. Therefore, it is highly desirable to develop a non-invasive method for the visualization of the initial water uptake site and tracking of water movement.

With scientific and technological advances, nuclear magnetic resonance (NMR) and magnetic resonance imaging (MRI) have become powerful tools in the study of seed water absorption and distribution in real time without damaging the seeds [2–4]. NMR enables detection of ^1^H protons, which are present primarily in water in the seed, and visualization of their spatial distribution, thus NMR spectrometry has been widely adopted as an analytical technique to provide visualized internal water information in the seed [5]. In addition, MRI has been used successfully in research on water uptake by seeds of *Oryza sativa* [6], *Solanum tuberosum* [7], *Lupinus luteus* [8], and *Tillia miqueliana* [9]. Therefore, MRI was deemed to be the most appropriate technique for studying seed imbibition.

*Gleditsia sinensis* (Caesalpiniaceae), also known as Chinese honeylocust, Taiwan tree, and hanging knife tree, is an economically valuable, deciduous tree used for landscaping in most regions of China. The wood is used for crafts and furniture, seed products are widely used in the food, cosmetic, industrial manufacturing, and pesticide industries, and the root bark exhibits anthelmintic and antifebrile properties.

*G. sinensis* is a unique longevity tree species in China. The optimal method for its propagation is by seeds, but the water-impermeable seed coat hinders germination. In recent years, the scale of artificial planting of *G. sinensis* has gradually increased, while there is a great shortage of good seed species and supporting cultivation techniques, which led us to further study the seed. To the best of our knowledge, the mechanism and anatomical characteristics of water absorption by *G. sinensis* seeds have not been investigated. Especially, visualizing mechanisms in seeds has always been a challenge for researchers due to the opaque nature of the seed coat [3]. In this study, seed imbibition without seed destruction was observed using MRI. Given the importance of understanding the relationship between water absorption and germination, the aims of this study were to describe the morphology and anatomy of the seed coat of *G. sinensis*, to identify the initial water uptake site and water movement using three methods (a dye-tracking test, MRI, and a blocking test), and to determine the relationship between seed structure and water absorption.

## Results

### Comparison of water imbibition between cracked and intact seeds

**Fig. 1:**
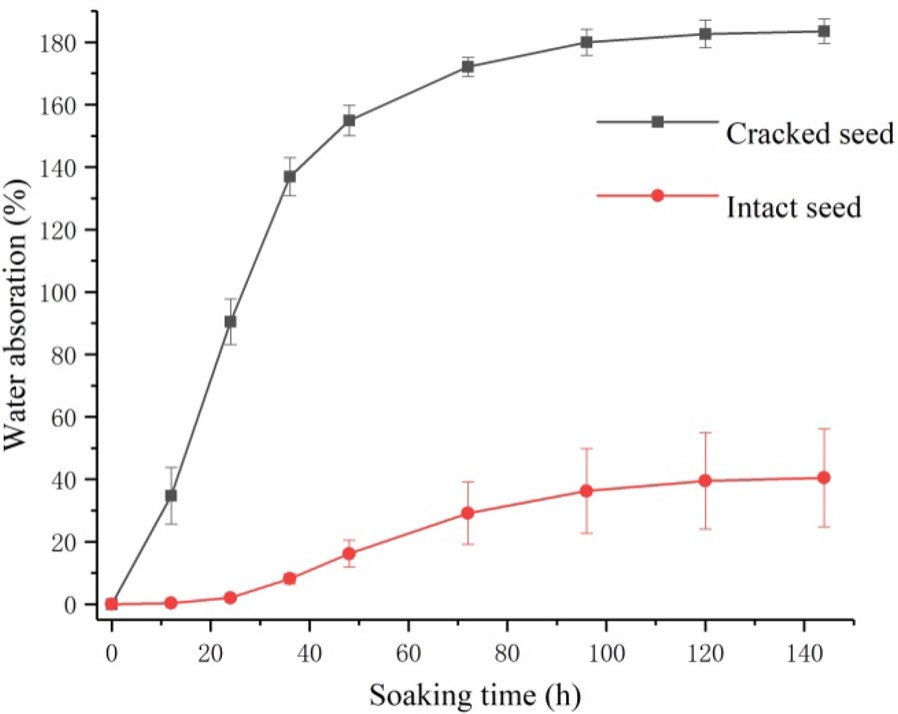
Water absorption curves for intact and manually cracked *Gleditsia sinensis* seeds with duration of soaking. Error bars represent the standard deviation

The increase in water absorption of manually cracked and intact (nontreated) seeds during imbibition was sigmoidal (Fig. 1). During the imbibition of cracked seeds, a rapid increase in water absorption was observed until 36 h when it attained 142.0%. Thereafter the rate of imbibition declined and the seed mass increased to 179.2% at 72 h and stabilized at 188.2% at 96 h. For the imbibition of intact seeds, water absorption attained 8.3% at 36 h and the rate of imbibition was substantially slower than that of manually cracked seeds. The final water absorption of intact seeds at 144 h was 30.3%, significantly lower than that of the cracked seeds (188.2%). These results showed that the intact seed coat strongly prevented imbibition of *G. sinensis* seeds.

### Effect of hot water treatment on seed coat impermeability

The impact of water temperature on seed coat impermeability is summarized in Table 1. The percentage of swollen seeds in the control group was 3.8%, which indicated that the hard seed coat strongly inhibited water absorption. The hot water treatments increased the permeability of the seed coat. With increase in water temperature, the percentage of swollen seeds increased significantly. The swollen percentage in the 70, 80, and 90 °C treatments increased significantly to 38.6%, 82.8%, and 91.4%, respectively, but the difference between the 80 and 90 °C treatments was not significant. The viability of the control seeds was 91.3%, which was higher than the viabilities of 89.3% and 87.3% when the seeds were immersed in water at 70 and 80 °C, respectively, but the differences in viabilities among these treatments and the control were nonsignificant. The viability of seeds soaked in 90 °C water plummeted to 57.0%. Thus, high water temperatures aided imbibition but an excessively high water temperature caused a degree of damage to the seeds. In this experiment, water with an initial temperature of 80 °C was optimal to break the seed coat impermeability of *G. sinensis* seeds.

**Table 1:** Effects of hot water treatments to break the seed coat impermeability on percentage of swollen seeds and viability of *Gleditsia sinensis* seeds

Treatments	Swollen percentage %	Viability%
Control	3.8 ± 2.1 c	91.3 ± 1.2 a
70 °C	38.6 ± 4.5 b	89.3 ± 3.1 a
80 °C	82.8 ± 3.0 a	87.3 ± 3.0 a
90 °C	91.4 ± 5.4 a	57.0 ± 1.0 b

Values (mean ± SD) within a column followed by the same letter are not significantly different (Duncan’s multiple range test, *p* ≤ 0.05)

### Ultramorphological characteristics of the seed coat

**Fig. 2:**
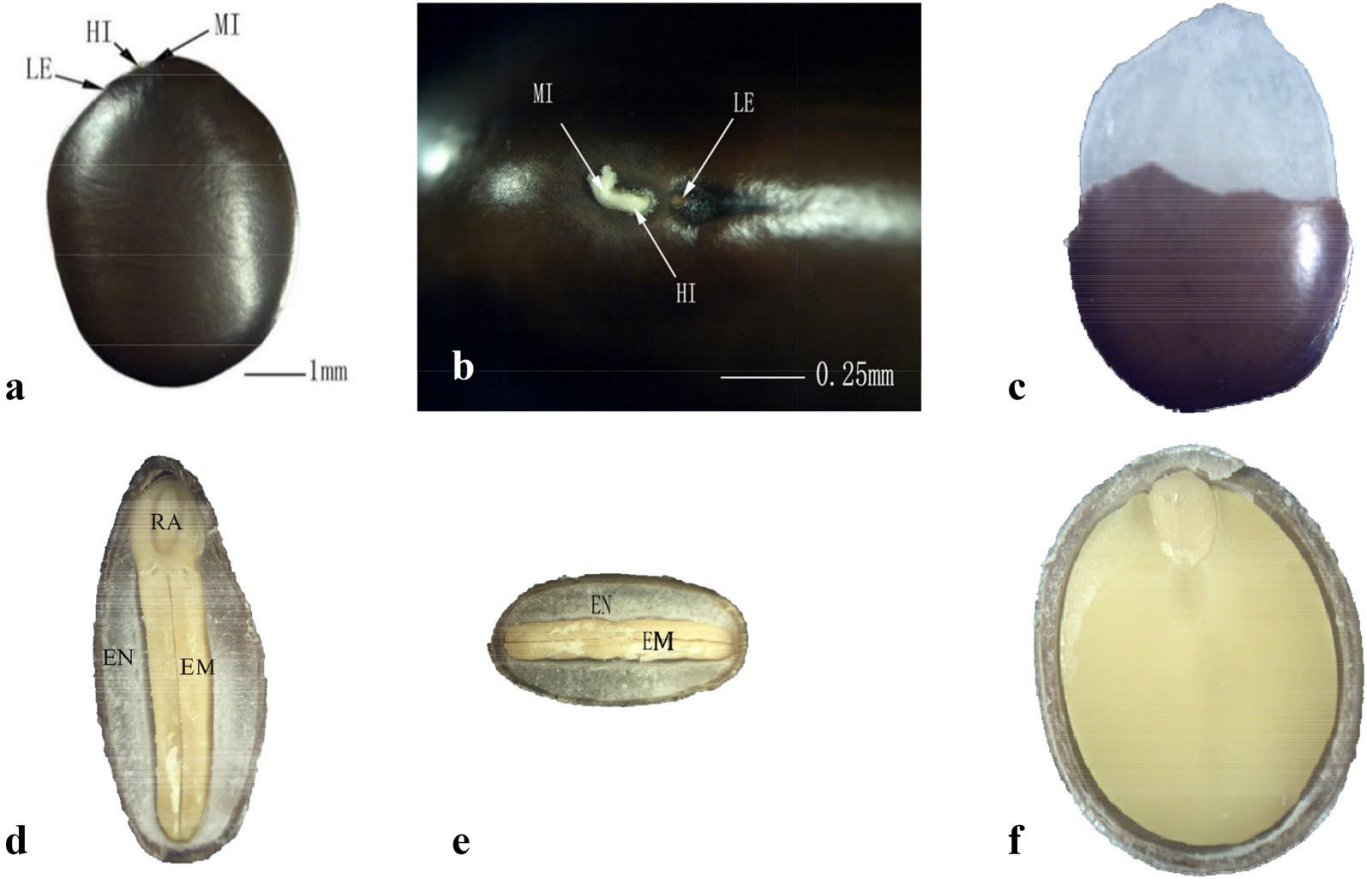
Structure of *Gleditsia sinensis* seeds viewed under a stereomicroscope. **A** a complete seed; **B** hilar region of the seed; **C** seed with seed coat partially removed; **D** longitudinal section of the micropyle (sagittal plane); **E** transverse section in the medial region of the seed (transverse plane); **F** longitudinal section of the seed (coronal plane). *EM* embryo, *EN* endosperm, *HI* hilum, *LE* lens, *MI* micropyle, *RA* radicle

Stereomicroscopic observation revealed that the *G. sinensis* seed shape is oblong with a distinct dorsoventral asymmetry. The hilar region is flat on one side of the seed and consists of the micropyle, hilum, and lens, which are linearly aligned (Fig. 2A). The entire seed, especially the micropyle and hilum, are covered with a thick layer of waxy substances (Fig. 2B), which can prevent water absorption of the seeds. Observation of the longitudinal section revealed that the embryo was encased by a thick nucellar-endosperm casing (Fig. 2C). The colloidal endosperm is distributed unevenly in the seed, with higher concentrations in the dorsoventral regions and lower concentrations in the two lateral sides, the radicle, and the chalazal region (Fig. 2D–F).

**Fig. 3:**
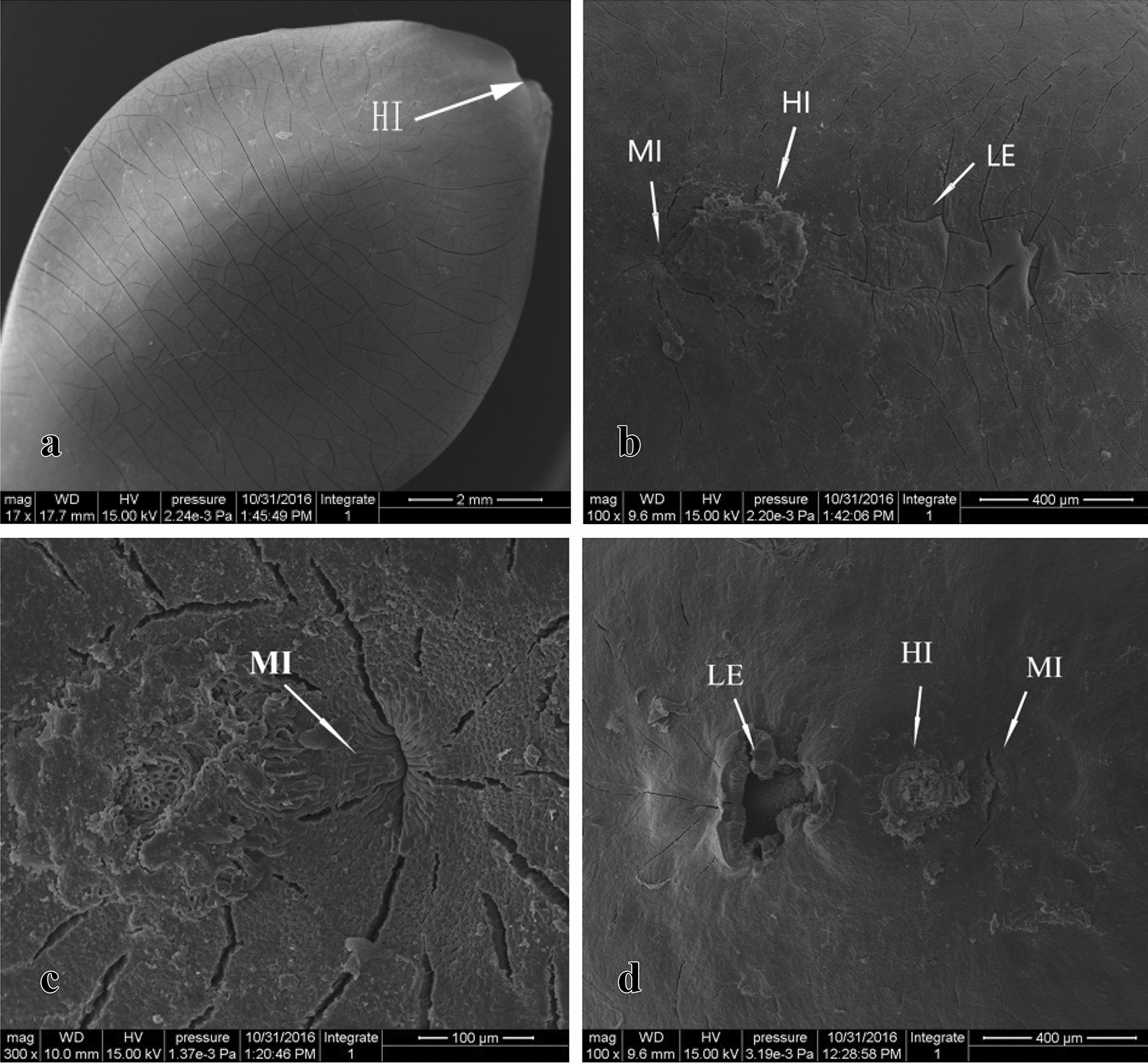
SEM images of the ultramorphology of *Gleditsia sinensis* seeds. **A** intact control seed (17×); **B** hilar region of a control seed (100×); **C** micropylar region of a mature seed (300×); **D** hilar region of a permeable seed (100×). *HI* hilum, *LE* lens, *MI* micropyle

SEM images of the seed coat surface revealed many intersecting vertical and horizontal cracks, including in the hilar region (Fig. 3A–C). After breaking seed coat impermeability, an area of the seed coat was detached at the lens (Fig. 3D). The seed coat cracks of permeable seeds were deeper than those of the control, and internal tissues were observed through the cracks (Fig. 3C, D). No other visible differences to the seed coat were observed between impermeable (control) and permeable seeds.

**Fig. 4:**
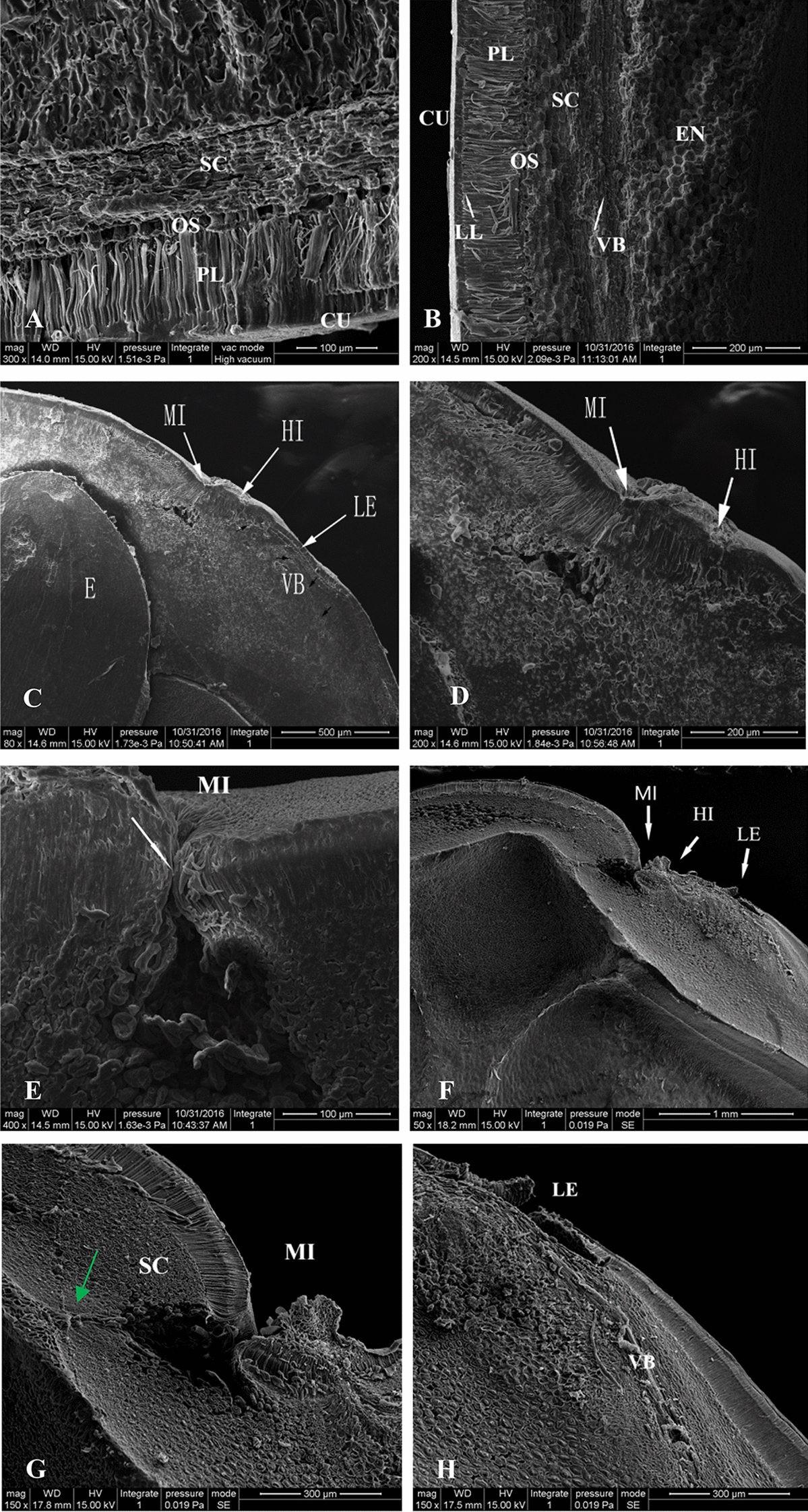
SEM images of the longitudinal and transverse structure of *Gleditsia sinensis* seeds. **A** transverse section of the seed outside the hilar region (300×); **B**–**D** longitudinal sections of mature seeds in the hilar region (80×, 200×, and 200×, respectively); **E** longitudinal section of mature seeds at the micropyle (400 ×); **F**–**H** longitudinal section of the seed in the hilar region (**F**), micropyle (**G**), and lens (**H**) after reduction in seed coat hardness (magnification 50×, 150×, and 150×, respectively). *CU* cuticle, *EN* endosperm, *HI* hilum, *LE* lens, *LL* light line, *MI* micropyle, *OS* osteosclereid layer, *PL* palisade layer, *SC* sclerenchymatous cell layer, *VB* vascular bundle

The anatomy of the seed coat can influence water imbibition. The seed coats in the three regions of mature (Fig. 4A–E) and permeable (Fig. 4F–H) seeds were observed by SEM. The SEM images illustrated that the majority of the seed coat, from the outer to the inner surfaces, consisted of the cuticle, palisade layer, osteosclereids, and several layers of sclerenchymatous cells (Fig. 4A). The cuticle is the outermost layer that limits water entry into the seed and was ~ 15 μm thick. The palisade cell layer beneath the cuticle consisted of a single cell layer ~ 60 μm in height. Between the palisade layer and cuticle was a continuous light line (Fig. 4A, B). Internal to the palisade layer was a layer of osteosclereid cells arranged perpendicular to the palisade cells. The sclerenchymatous cell layer, located beneath the osteosclereids, was composed of multiple layers of tightly packed cells. In addition to these four layers, an additional structure of a vascular bundle extended from the hilar region to the lens, and continued beneath the sclerenchymatous cell layer (Fig. 4B–D). In the micropyle, a weakly discernible marking line separated the palisade cells into two parts (Fig. 4E, indicated by white arrow).

Hot water treatment caused the tightly closed micropyle to open (Fig. 4F) and the cavity under the micropyle increased in size (Fig. 4C–G). In the middle parts of the sclerenchymatous some layer of cells became more loosened than in the control, especially those away from the micropylar side (Fig. 4C, F). A linear narrow channel which connected the lower side of the cavity and the inside of the seed coat was found (indicated by green arrow, Fig. 4G). The cuticle layer ruptured near the hilar region and the layer of palisade cells in the lens was raised (Fig. 4H).

### Tracking the imbibition pathway by TTC staining

Dye-tracking tests, such as TZ staining [10], can be used to locate the site of water entry into a seed [11]. The principle of TZ staining is that the dehydrogenases responsible for the staining reduction are only active in the living cells of imbibed seeds. When a colorless TTC solution is imbibed by the seed, it will accept hydrogen from the dehydrogenases, producing a red, stable, and non-diffusible substance. The non-imbibed tissues cannot stain red, even though the cells are living, thus the color change in a viable seed distinguishes the tissues that have been imbibed.

**Fig. 5:**
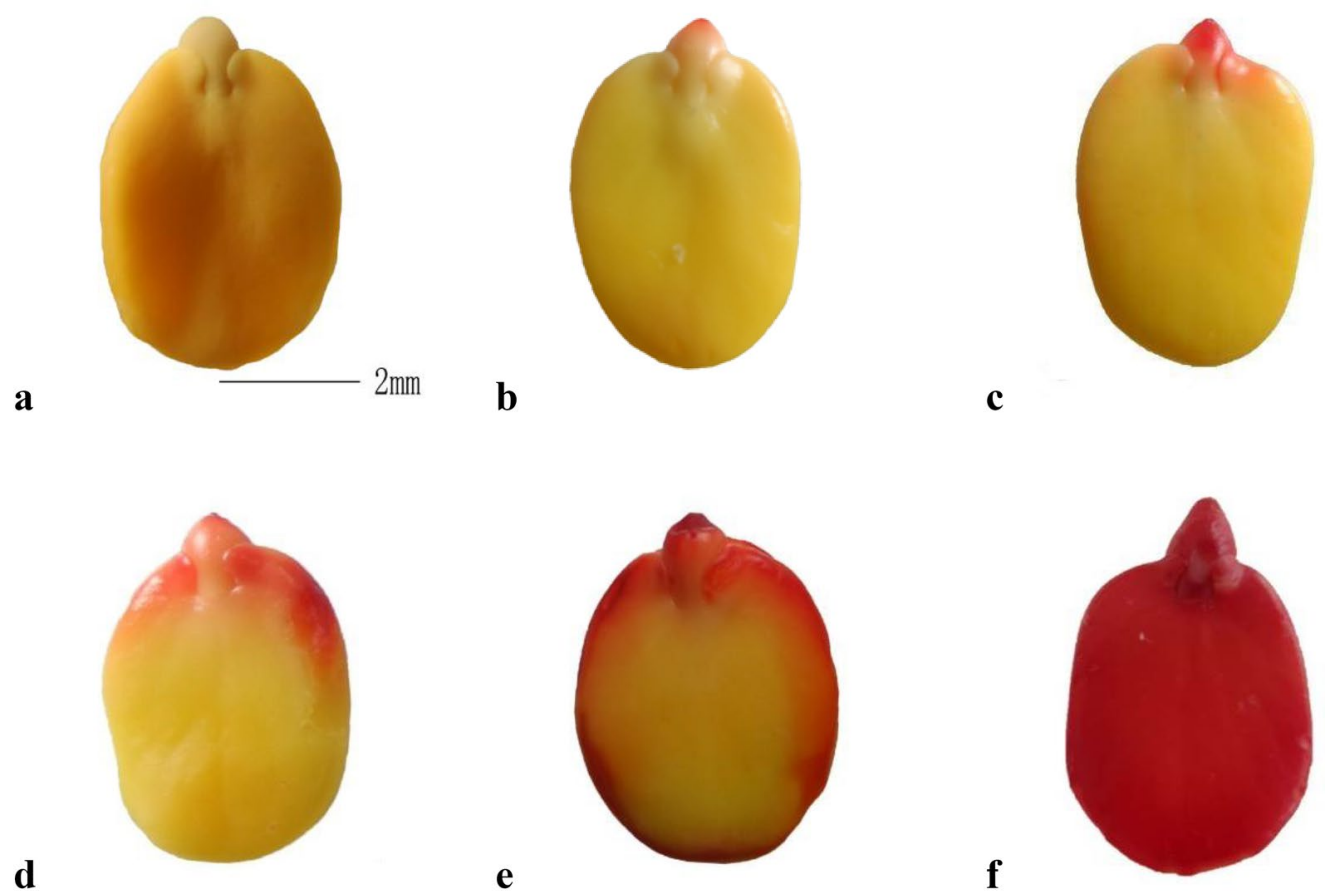
TTC staining during imbibition of *Gleditsia sinensis* seeds. **A** control seed; **B** staining after 6 h; **C** staining after 12 h; **D** staining after 24 h; **E** staining after 48 h; **F** staining after 72 h

As indicated by the control permeable seed (Fig. 5A), the embryo color before dying was yellow. After 6 h of incubation in TTC solution, red staining was first observed at the tip of the radicle (Fig. 5B). After staining for 12 h, the entire radicle and the right side of the adaxial cotyledon (which is under the lens) were stained red, whereas the left side was not stained red (Fig. 5C). Subsequently, red staining expanded along each lateral side to the chalazal of the cotyledons (Fig. 5D), and the red-stained area of the cotyledon on the hilar side (right side) was significantly larger than that on the other side. At 48 h, the entire lateral region of the cotyledon was stained and the stain area gradually penetrated to the central axis of the cotyledon, but the majority of the central cells of the cotyledon were unstained (Fig. 5E). After sufficient imbibition, the radicle elongated slightly and the entire embryo was stained red (Fig. 5F). These results indicated that the initial site of water absorption was the tip of the radicle. The direction of water migration in the seeds was from the radicle towards the cotyledon apices along the lateral sides of the cotyledons. Subsequently, water entered the central cells of the cotyledons. After 12 h, the first area of the cotyledon to be stained was on the left side, but the red-stained area on the right side of the cotyledon was more extensive than that on the left side after staining for 24–48 h. This trend can be explained by the presence of a vascular bundle on the side of the hilar region.

### MRI analysis of water absorption

**Fig. 6:**
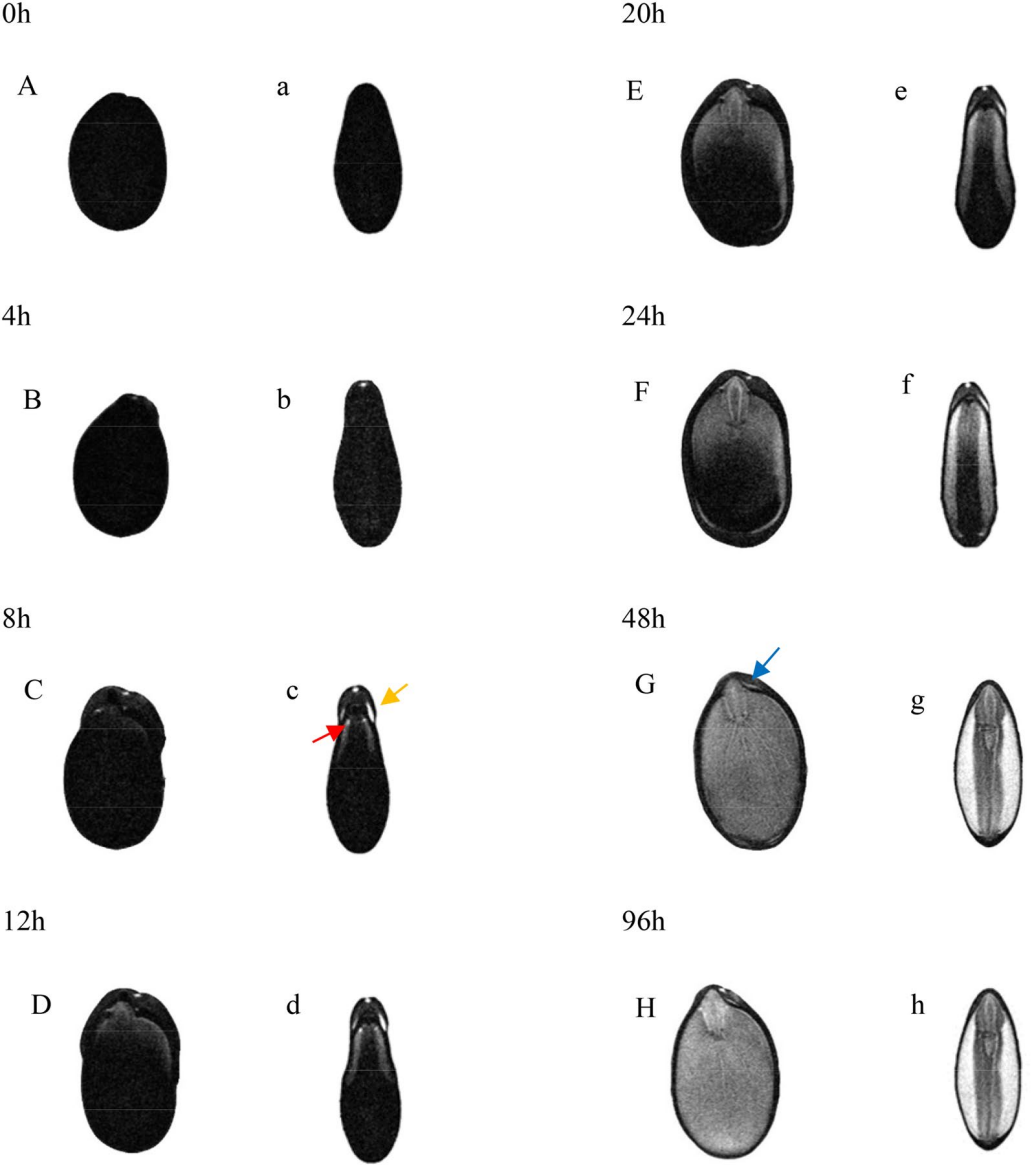
Magnetic resonance imaging maps of mature *Gleditsia sinensis* seed (**A**, **a**) and seeds imbibing for 0, 4, 8, 12, 20, 24, 48, and 96 h (**B**–**H**, **b**–**h**) in two sections, the capital letters represent the coronal planes and the lowercase letters represent the sagittal planes

MRI provides an approach for non-invasive detection of the spatiotemporal distribution of water during seed imbibition [2, 6, 12]. The principle is that MRI employs static magnetic fields and radio frequencies to obtain images of proton mobility in biological systems. With regard to the grayscale map, the higher the density of ^1^H protons, the brighter the image [2]. During imbibition the seed volume increased significantly, so 13–20 layers of the seeds at each stage were scanned by MRI. And in this paper, for each stage, the seed only showed two layers (coronal and sagittal plane) of MRI images (Fig. 6). In the mature seed (0 h), the seed was presented as black (Fig. 6A, a) which inferred that the water content or the hydrogen proton count was extremely low. After 4 h imbibition, a bright spot at the cavity region under the micropyle appeared (Fig. 6b), which suggested that the initial water absorption site was the micropyle. After imbibition for 8 h, the seed coat of the hilar region was imbibed noticeably (Fig. 6C) and the bright area of the right side (indicated by a yellow arrow) was larger and brighter than that on the left side. According to the anatomy of the seed coat, the brighter parts are the narrow space between seed coat and endosperm. Also, at this time the intersection of the radicle tip, the endosperm, and the cotyledon became brighter (indicated by a red arrow). At 12 h, the intensity of water signal of the entire radicle increased significantly. Meanwhile, the signal at the intersection of the endosperm and the cotyledon was enhanced further. Therefore, for *G. sinensis* seeds, after water entered the seeds through the micropyle, two water movement paths were found: migration along the space between the seed coat and the endosperm to the chalazal (Path I); migration from the embryonic axis to the cotyledons (Path II) with considerable swelling of the seeds. Up to 20 h, in the right side of the intersection between the endosperm and the cotyledons, the speed of water movement was still significantly quicker than that of the other side since a bright signal was observed in a larger area at the chalazal in the right side, as seen from Fig. 6E. From this map it also showed that the embryonic axis exhibited very strong signals, but water migration through Path II was far slower than that through Path I because most part of the image of the cotyledon was black. At 24 h, the signals at the intersection between endosperm and the cotyledons of both sides had arrived to the chalazal. When the seed was imbibed for 48 h, the plumule had been elongated as shown in the picture (Fig. 6g). The vascular is clearly shown in the seed coat (indicated by a blue arrow) for the exhibited bright signal from Fig. 6G. Before 48 h, the vascular should have exhibited a bright signal but this phenomenon could not be confirmed during scanning. After imbibition for 96 h (Fig. 6H, h), water continued to enter into the cotyledons, and the cotyledons were brighter than before, but the seed coat was still black.

### Water absorption by blocking different regions of the seed

**Fig. 7:**
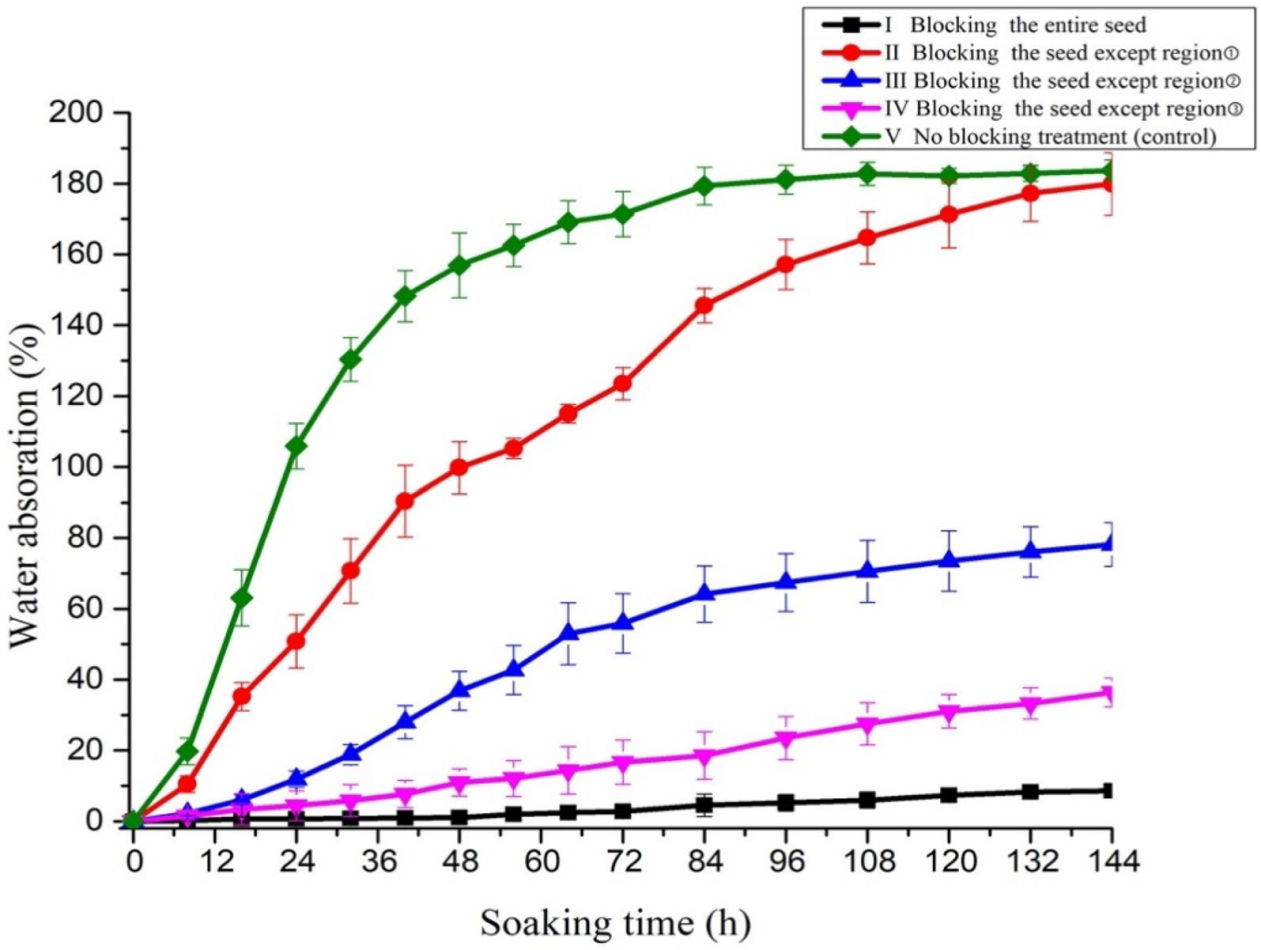
Water absorption curves for different regions of *Gleditsia sinensis* seeds. Error bars represent the standard deviation

The water absorption rate in treatment I (seed coat surface was blocked and submergence in water for 144 h) was 8.6%, which was considerably lower than that of the control (Fig. 7). Thus, Vaseline® effectively prevented seeds from absorbing water. Therefore, it was feasible to determine water absorption in different regions of the *G. sinensis* seed coat by applying Vaseline® to block water uptake in specific regions.

The water uptake rate in treatment V (no blocking) was significantly higher than that of the three blocking treatments throughout the imbibition period. At 8 h, seed water absorption in treatment V attained 19.8%, which was significantly higher than that of treatment II (10.5%). The increase in water absorption in treatments III and IV (2.1% and 1.5%, respectively) was significantly lower than that of treatment II. These results indicated that the hilar region was the initial site for water absorption. At up to 32 h, seeds of treatment V maintained the most water absorbed of 130.4%. Absorption in treatment II also increased rapidly to 70.7%. The absorption rates of treatments III and IV were 18.8% and 5.8%, respectively, which differed significantly. Thus, the rate of water absorption in treatment III increased more rapidly than that of treatment IV. Therefore, after the initial main phase of water absorption in the hilar region, the medial region of the seed began to uptake water, whereas little water was absorbed by the distal region. By 84 h, the seeds in treatment V attained a plateau phase and the amount of water absorbed was 179.3%. Seeds in treatment II attained the plateau phase at 144 h with water absorption of 179.9%, which was similar to the final percentage observed for treatment V. Water absorption in treatments III and IV attained 78.1% and 36.35%, respectively, at 144 h.

For the blocking treatments II, III, and IV, the initial stages of significant water absorption (when water absorption differed significantly from its former result) were at 8, 24, and 48 h, respectively. The results all pointed to the fact that different regions of the seed played different roles in the imbibition process.

## Discussion

### Breaking physical dormancy of seeds by hot water treatment

Seeds of the majority of Leguminosae species show hardseededness, with the seed coat being the main barrier for water uptake. Several methods, such as acid scarification, hot water treatment, and mechanical scarification [13–15], can be used to break the hardness of the seeds of such species. However, acid scarification can be environmentally detrimental and dangerous to the experimenter. Normally, hot water treatment is an efficient method for breaking the hard seed coat of some species and may be used effectively in propagation. For example, Patane et al*.* observed that hot water treatment increased germination in *Astragalus hamosus*, and that a water temperature exceeding 80 °C was lethal to seeds [16]. In the present experiment, the optimal treatment to break the seed coat impermeability of *G. sinensis* seeds was soaking in water at an initial temperature of 80 °C for 5 min. It was also concluded that seed viability was sensitive to water temperature. Kang proposed that 100 °C hot water treatment was the optimal temperature to break hardseededness and the seed still showed a high germination rate [17]. However, in the present study, 90 °C treatment reduced seed viability to 57.0%.

### Seed coat structure and its relation to water absorption

Stereomicroscopic observation revealed that the entire seed coat of *G. sinensis* was covered with waxy substances that may prevent water from entering the seeds. The hilum region of mature *G. sinensis* seeds was coated with a thicker layer of wax and the micropyle was sealed. These characteristics may be responsible for the impermeability of the seed coat. After hot water treatment, SEM images showed that the wax in the hilum region was reduced or disappeared. The micropyle was opened and cracks in the seed coat were wider and deeper (Fig. 4E), and the cuticle in the hilar region was ruptured. These structural changes may improve the permeability for seed imbibition. An interesting phenomenon observed in the present study was that a large piece of the seed coat at the lens had detached and the layer of palisade cells at the lens was raised (Fig. 4F). Although the degree of damage to the lens was more severe than that to the micropyle, MRI maps showed that the lens of *G. sinensis* seeds was not the first site to imbibe water. Thus, the water-gap could not be identified from morphological changes by itself in the seed coat after physical dormancy was broken.

Structural studies of impermeable seeds are important to better understand the causes and suitable dormancy release methods when subjected to treatments for dormancy release [18, 19]. Previous research has shown that water impermeability is due to the palisade layer [20]. According to Geisler et al*.* [21], resistance to water absorption by Leguminosae seeds is primarily due to water-impermeable substances in the palisade cells. Baskin [22] stated that the heavily lignified cell walls in the palisade layer may also render the seed impermeable to water. In some species, the palisade layer of the seed coat contains a light line, which is comprised of a high concentration of callose, a water-repellent substance that also contributes to seed coat impermeability [19, 21, 22]. Therefore, the presence of the palisade layer and the light line may be associated with the hardseededness and impermeability of the seed coat of *G. sinensis*.

In the present study, a vascular bundle, a distinctive structure in Leguminosae seeds, was observed in *G. sinensis* seeds that connected the hilum to the lens. de Paula et al*.* [23] concluded that the vascular bundle was associated with movement of water in *Senna macranthera* seeds*.* A vascular bundle was also reported in seeds of *Lupinus luteus* [8] and *S. multijuga* [19]. From the maps of anatomy, it can be speculated that some other structures are also beneficial for water migration within permeable seed. For example the sclerenchymatous cells becomes loose in the left side of the seed around the micropyle. Between the lower side of the micropyle cavity and the inner seed coat, a linear channel exists, which may be helpful for water enter into radicle.

### Location of water entry and the pathway of water movement in the seeds after reduction of seed coat hardness

Dye-tracking experiments like the TZ test have been used to identify the migration of water in the seeds of some plants. In this study, it was found that the radicle was the first region to stain red. The radicle is adjacent to the micropyle, therefore it was suspected that the initial imbibition site of *G. sinensis* seeds was the micropyle. However, owing to methodological limitations, water movement in the seed coat and endosperm cannot be assessed directly. A fundamental basis of the TZ test is that only living cells can stain red. The seed coat and colloidal substances of the endosperm cannot stain, thus water movement in the seed coat and endosperm could not be detected. In addition, this method is labor-intensive, time-consuming, destructive, and requires monitoring several seeds to allow for variation among seeds.

The MRI technique, which is a non-destructive, real-time visualization technology, effectively overcomes the aforementioned shortcomings by acquiring spatial and proton mobility information in biological systems [24]. The technology has been widely used to study seed imbibition [8, 25–27]. In the present research, the MRI maps acquired at 4 h imbibition (Fig. 6-b) clearly indicated that the micropyle was the initial site for water absorption in the *G. sinensis* seed. This finding confirmed that our hypothesis based on the TZ staining was correct. de Paula et al*.* [23] reported that water entered thermally scarified seeds of *Cassia leptophylla* through the micropylar canal. However, in scarified *Erythrina speciosa* seeds, water first entered the seed through the hilum [28, 29], and Jaganathan et al*.* [29] confirmed that water entered seeds only through the lens in *Delonix regia*. These results indicated that the primary site of water entry into seeds after permeabilization of the seed coat varied among species.

The MRI maps also enabled us to determine water movement in *G. sinensis* seeds. When water entered into the seed, the signal strength was enhanced gradually. The scanner maps at 8 h indicated that water entered into the seed from the micropyle, was stored in the cavity, then migrated through the narrow channel indicated by the green arrow in Fig. 4G, and finally split along two paths where some water migrated along the Path I to the chalazal region and the other water reached the embryonic axis and spread into the cotyledons through Path II.

MRI and TZ test showed that water migration in the hilar side was faster than on the other side of the seed (Fig. 5, 6D). These findings clearly certified that the vascular bundle is an important structure for water movement. In addition to the seed coat and embryo, *G. sinensis* seeds contain a colloidal endosperm which is composed predominantly of galactomannan [30]. The MRI maps at 24 h revealed that the bright signal of ^1^H protons in the endosperm had reached the chalazal region, but nearly half of the cotyledons did not exhibit water signal. Thus, the endosperm may prevent water from entering the seed and hinder the early imbibition process.

The quantitative results of the blocking experiment confirmed that the hilar region played an important role in imbibition by water absorption during the initial 8 h. Based on the results in treatment III and IV, the medial region and the bottom of the chalazal region also showed capability for imbibition at 144 h, however, water absorption was substantially less than that of the hilum region. In *G. sinensis* seeds, the micropyle, hilum, and lens are in overly close proximity to differentiate with the naked eye and reliably block them individually. From the presently applied methods it can be concluded that initial imbibition occurred in the hilar region, but the structure that is the primary water-gap for water absorption could not be quantified.

Three methods of identification of the route of water entry into seeds of *G. sinensis* seeds were used in this study, however, none of these studies clearly documented water movement in the seed coat, and thus further study is required.

## Conclusion

In our study it was found that 80 °C water was optimal to break hardseededness of *G. sinensis*, which is an effective and convenient method. SEM images revealed that the palisade layer and light line in the seed coat can hinder water entry into the seed. When hardseededness was broken, the micropyle was the initial site for water imbibition. Water entered the seed through the micropylar cavity, then migrated through the narrow channel, and finally separated into two paths where some water migrated along Path I, and the other water migrated along Path II. By the quantitative results of the blocking experiment, it was inferred that the medial region and chalazal portion were capable of imbibing water when the hilar region was blocked in nature, but water absorption occurred later and was slower than that through the hilar region. Combined with other studies the relation between seed coat structure and water imbibition can be concluded. Furthermore, MRI can provide a non-destructive, real-time visualization technology for seed science.

## Materials and methods

### Plant material

*Gleditsia sinensis* seeds were collected from Yan’an City, Shaanxi Province, China. The seeds were soaked in water to separate empty and insect-damaged seeds. The remaining seeds were dried at room temperature and stored at 4 °C. The weight of 1000 seeds were 186.06 g and the viability was 93%.

### Water absorption curves

Water absorption was calculated for intact and cracked (slit at the bottom of the cotyledons) seeds and plotted to compare water absorption between the two treatments. Three replicates of 30 seeds were used for each treatment. Seeds in each treatment were weighed initially to the nearest 0.001 g (*m*) and immersed in water in a beaker. The seeds were removed from the beaker, wiped with absorbent paper to remove excess water on the seed surface, weighed (*m*_1_) and returned to the beaker. This process was repeated at 12 h intervals up to 48 h and then at 24 h intervals until the plateau phase was attained. This experiment was conducted at room temperature. The equation used to calculate water absorption was as follows:

$${\text{Absorption }}\left( \% \right) = \frac{{m_{1} - m}}{m} \times 100$$

where *m* and *m*_*1*_ represent the seed mass (in grams) before and after soaking in water, respectively.

### Breaking the seed coat impermeability

To break the impermeability of the seed coat, seeds were treated by immersion in a beaker of water placed in a heat bath maintained at 70, 80, or 90 °C for 5 min [29]. After treatment, the beaker was cooled to room temperature and the seeds soaked for 24 h. The control seeds were soaked in water at room temperature for 24 h. In this experiment, the treatments were applied with four replicates of 100 seeds each. The treatment efficacy was evaluated by recording the percentage of swollen seeds and the seed viability, which was determined using the tetrazolium (TZ) test. For the viability test, imbibed seeds were used; hard-coated seeds needed to be mechanically cut at the bottom of the cotyledons to make the seeds permeable. The entire seed coat was removed and the embryo was excised. Subsequently, the embryos were stained in 0.5% 2,3,5-triphenyltetrazolium chloride (TTC) [10].at 35 °C in the dark for 5 h. After staining, the viability of the seeds was determined in accordance with the International Seed Testing Association rules [10].

### Observation of seed coat surface and anatomy

The seed coat surface was observed under a stereomicroscope (SZX16, Olympus, Tokyo, Japan) to determine its ultramorphological features, including the surface characteristics of the seed coat, longitudinal sections through the lens and micropyle, and transverse section through the medial region of the seed. A scanning electron microscope (Quanta 200, FEI, Portland, OR, USA) was used to observe the microstructure of the seed coat, especially in the hilar region. Nontreated seeds (control) and permeable seeds (soaked in 80 °C water for 5 min, which was the optimal treatment to break seed coat impermeability in the preceding experiment) were sampled and the seed coat surface, transverse sections, and longitudinal sections of the seeds were prepared by cutting through the hilum region. The samples were first fixed with formaldehyde–acetic acid–alcohol solution, then dried by critical-point drying. Small blocks of the specimens (about 3 mm in length) were observed under the SEM operated at 15 and 20 kV at 0.45–0.68 Torr.

### Dye-tracking of imbibition pathway

Staining with TTC was used for a dye-tracking experiment to trace the movement of water through the permeable seed coat (treated with 80 °C water for 5 min) during imbibition. In this experiment, 80 permeable seeds were placed in 0.5% TTC solution and incubated at 35 °C in the dark [10]. Ten seeds were removed from the solution at 6 h intervals and rinsed with tap water. The seed coat and endosperm (which are not dyed red for they are not living tissues) were removed to observe the stain intensity of the embryo and identify the seed imbibition pathway. This procedure was continued until the embryos had been stained completely.

### MRI measurement of water absorption

The MRI analysis was conducted using a high-field NMR analyzer (PharmaScan, Bruker Biospin GmbH, Germany) equipped with a 38-mm-volume coil and 7.0 T permanent magnet corresponding to a proton resonance frequency of 300.337 MHz at 22 ± 1 °C. MRI scans were performed on the intact seeds. The first scan was performed a seed that did not undergo hot water treatment for control. The seed was then soaked in 80 °C water for 5 min. The NMR scans were performed after 0, 4, 8, 12, 20, 24, 48 and 96 h. The images were recorded in two planes. Anatomical images were captured with a turbo-rapid acquisition relaxation enhancement (RARE) PD-weighted sequence (repetition time (TR)/echo time (TE) = 2345.5/13 ms, slices = 20, field of view (FOV) = 2.8 × 2.8 cm, number of averages = 20, matrix = 256 × 256, slice thickness/gap = 0.4/0 mm, flip angle = 180°), scan time = 13 min, and pixel resolution = 109 μm.

### Water uptake by blocking different seed regions

**Fig. 8:**
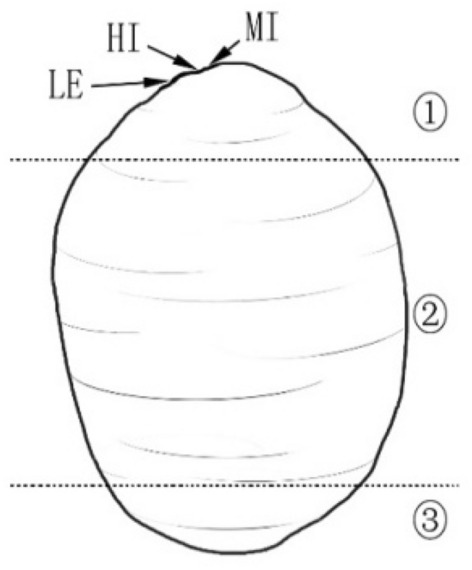
Schematic diagram of *Gleditsia sinensis* seed regions. The seed was divided into three regions: the hilar region including the hilum, micropyle, and lens ①, the medial region ②, and the chalazal region (apex of the cotyledons in the seed) ③, HI, hilum; LE, lens; MI, micropyle

To measure the water absorption capability of different regions of the seed (Fig. 8), Vaseline® was used to make the permeable seed coat partially impermeable [29, 31–33] and the water absorption was quantified by determining the changes in weight. In this experiment, the seeds were first soaked in 80 °C water for 5 min to break the seed coat impermeability, then four replicates of 30 seeds each were weighed to determine the initial seed weight. Subsequently, Vaseline® was used to partially block the seed coat in the following four treatments as listed in Table 2. No Vaseline® was applied to the control seeds. After treatment, all seeds were soaked in 150 mL distilled water at room temperature. At 8 h intervals, the soaked seeds were removed from the water and wiped to remove exterior water and the Vaseline® completely. The samples were weighed as before. Vaseline® was then reapplied to the same original region (except the control seeds) and returned to the water. This process was repeated until 144 h (when the control group attained saturation).

**Table 2:** Treatments to block water uptake in three regions of *Gleditsia sinensis* seeds

No	Treatments
I	Blocking the entire seed
II	Blocking the seed except region ①
III	Blocking the seed except region ②
IV	Blocking the seed except region ③
V	No blocking treatment (control)

### Statistical analysis

For each assay, the summary statistics for the data are presented as the mean ± standard error (SE). Duncan’s multiple range test, as implemented in IBM SPSS Statistics 19.0 software (IBM, Armonk, NY, USA) was used for all data analyses, with *p* < 0.05 applied as the threshold significance level. Data were plotted using Origin 8.5 software (OriginLab Corporation, Northampton, MA, USA). The MRI images were examined using Bruker ParaVision 5.1 software (Bruker, Germany).

## Data Availability

Not applicable.
